# Barriers Experienced by Psychiatric Nurses to Facilitate Therapeutic Relationships With Mental Healthcare Users With Dual Diagnosis in a Psychiatric Hospital in South Africa

**DOI:** 10.1111/jpm.13136

**Published:** 2024-11-18

**Authors:** Thomas Mathebula, Anna Elizabeth van der Wath, Thifhelimbilu Irene Ramavhoya

**Affiliations:** ^1^ Department of Nursing Science, Faculty of Health Sciences University of Pretoria Pretoria South Africa; ^2^ Department of Nursing Science, Faculty of Health Sciences University of Limpopo Mankweng South Africa

**Keywords:** dual diagnosis, mental healthcare users, psychiatric nurses, substance use disorders

## Abstract

**Background:**

Psychiatric nurses are in a unique position to build therapeutic relationships with mental healthcare users with dual diagnoses to foster trust and recovery. However, a dual diagnosis poses barriers to establishing and maintaining a therapeutic nurse–patient relationship.

**Aim:**

The overall aim of this study was to explore and describe barriers experienced by psychiatric nurses to facilitate therapeutic relationships with mental healthcare users with dual diagnosis in a psychiatric hospital in Limpopo province, South Africa.

**Design:**

A qualitative, explorative, descriptive and contextual design was followed.

**Method:**

Semi‐structured interviews were conducted with 12 purposively selected participants who cared for mental healthcare users with dual diagnoses. Tech's method of open coding was used to analyse the data.

**Findings:**

Disruptive and disrespectful behaviour and mental healthcare users' substance use during hospitalisation resulted in nurses harbouring ambivalent feelings that impaired therapeutic nurse–patient relationships.

**Conclusion:**

The barriers affecting therapeutic relationships with mental healthcare users with dual diagnosis should be addressed to enhance recovery and treatment compliance.

**Recommendations:**

An integrated approach with the involvement of the interprofessional team, debriefing and training for nurses may help to foster therapeutic nurse–patient relationships, empower nurses and enhance the recovery of mental healthcare users with dual diagnosis.

**Summary**

- What is known on the subject?
  ○ Mental healthcare users with substance use (dual diagnosis) often present with social, psychological and physical complications that might affect access to care and treatment compliance.
  ○ Psychiatric nurses play a critical role in the management of mental healthcare users with dual diagnosis.
  ○ Although therapeutic nurse–patient relationships significantly improve patient outcomes, nurses experience difficulties in establishing relationships with mental healthcare users with dual diagnoses.
- What this paper adds to existing knowledge?
  ○ Mental healthcare users' disruptive and disrespectful behaviour and substance use during hospitalisation form barriers to establishing therapeutic relationships.
  ○ Nurse–patient relationships characterised by ambivalent feelings may affect the recovery process.
- What are the implications for practice?
  ○ Integrated interprofessional interventions are required to help mental healthcare users acquire coping strategies to overcome substance use.
  ○ Debriefing and training for nurses may help to foster therapeutic nurse–patient relationships, empower nurses and enhance the recovery of mental healthcare users with dual diagnosis.

## 1 Introduction

Dual diagnosis refers to the comorbidity between substance use and a psychiatric disorder, of which the latter is often a psychotic or affective disorder (Adan and Benaiges 2016). Compared to adults with only one type of diagnosis, those with dual diagnosis experience significantly more socio‐behavioural problems (Jegede et al. 2022). Dual diagnosis is associated with social, psychological and physical complications that might affect access to care and treatment compliance (Chiappini et al. 2024).

There is a high prevalence of psychiatric comorbidity among patients with substance use (Daigre et al. 2019). ‘About one third of people with a substance use condition also experience a mental health condition, and people with a mental health condition are also more likely to develop a substance use condition’ World Health Organization (2022: 7). A study in the USA estimated that adults with dual diagnosis constituted 25.8% of those with any psychiatric disorder (Jegede et al. 2022). Despite the high prevalence, dual diagnosis is often underdiagnosed and not effectively managed (Fantuzzi and Mezzina 2020).

Lafleur‐Omeler (2023) state that patients with substance use disorders in psychiatric units require specialised care and management to address their physical, mental and emotional needs to prevent relapses, decrease hospital readmissions and improve their quality of life. Management of mental healthcare users (MHCUs) with dual diagnosis can be challenging due to the interaction between psychoactive substances and mental illness that may affect treatment and clinical outcomes (Chiappini et al. 2024). Dual diagnosis is one of the leading causes of disability globally and a failure to identify and address the complex healthcare needs of these MHCUs affects relapse rates and contributes to the burden of the disease (Anandan, Cross, and Olasoji 2024).

Various barriers exist in low‐ and middle‐income countries to access timeous treatment of substance use disorders such as reluctance to seek help due to fear of stigma or normative cultural views on substance use (Heijdra Suasnabar and Hipple Walters 2020). Once MHCUs with dual diagnoses are admitted to psychiatric facilities, nurses face multiple challenges in providing safe and high‐quality care (Lafleur‐Omeler 2023). These challenges include high staff turnover, lack of competency (Lafleur‐Omeler 2023), barriers to developing empathy with MHCUs with dual diagnosis (Anandan, Cross, and Olasoji 2024) and lack of self‐confidence, doubts and concerns (Anandan et al. 2024).

The authors identified some gaps in the area of dual diagnosis research. Guidelines for substance use disorders and serious mental illness lack clear strategies for the diagnosis, treatment and management of dual disorders. There is a need to improve person‐centred and integrated care for MHCUs with dual diagnosis (Alsuhaibani et al. 2021). Mental health professionals base their management of MHCUs with dual diagnosis on their attitudes towards substance use which were judgmental in some cases (Pinderup 2018). Research to address attitudinal barriers may help to understand the reasons for the stigmatisation of MHCUs with dual diagnosis by healthcare professionals and how it could be overcome (Mthombeni 2021). Further research is also needed on nurses' self‐efficacy in caring for MHCUs with dual diagnoses (Anandan et al. 2024).

Nurses play a critical role in the management of MHCUs with dual diagnosis. Helpful nursing care from the perspective of mental health inpatients with a dual diagnosis includes among others the building of a therapeutic relationship (Brahim et al. 2020). Therapeutic nurse–patient relationships are essential in mental health nursing and significantly improve patient outcomes (Girard et al. 2021). A therapeutic relationship enables nurses to facilitate change during the recovery period (Wright 2021) and improve person‐centred care in mental health nursing (Aznar‐Huerta et al. 2021). Specific research on therapeutic relationships in acute psychiatric settings is limited and efforts to raise awareness of potential barriers may increase the chances of improving nurse–patient relationships (Bolsinger et al. 2020). Barriers in the nurse–patient therapeutic relationship include nurse‐related, patient‐related and organisation‐related barriers. Nurse‐related barriers are reflected in inadequate skills, work exhaustion, and negative attitudes of nurses (Pazargadi et al. 2015). Patient‐related barriers include power imbalances and feelings of insecurity and being dehumanised during inpatient treatment (Bacha, Hanley, and Winter 2020).

In this study, the researchers were interested in understanding the barriers experienced by psychiatric nurses to facilitate therapeutic relationships with MHCUs with dual diagnosis in a psychiatric hospital in Limpopo Province, South Africa. The shortage of mental healthcare specialists and dilapidated infrastructure in psychiatric hospitals in Limpopo Province compromise staff and patient safety and privacy (Manganye 2021). The first author (a psychiatric nurse) observed that psychiatric nurses struggled to maintain structure in the hospital while striving to develop caring and trustful relationships with MHCUs. Nurses sometimes respond angrily to unruly MHCUs and their colleagues' responses to such behaviour; feelings that may affect the treatment provided to MHCUs.

### 1.1 Aim

To explore and describe the barriers experienced by psychiatric nurses to facilitate therapeutic relationships with MHCUs with dual diagnosis in a psychiatric hospital in Limpopo Province. The findings informed recommendations on how to facilitate therapeutic relationships with MHCUs with dual diagnosis.

## 2 Method

### 2.1 Design

The qualitative and exploratory design (Polit and Beck 2021) provided an in‐depth description of the barriers experienced by psychiatric nurses to facilitate therapeutic relationships with MHCUs with dual diagnosis. The COREQ checklist recommendations for reporting were followed (Tong, Sainsbury, and Craig 2007).

### 2.2 Population and Setting

This research was conducted at a public psychiatric hospital with short‐ and long‐term wards, situated in the Mopani District of Limpopo Province. At the time of the study, there were six male wards, two female wards and one mixed ward for children. The short‐ and long‐term wards were staffed by 8 male nurses and 202 female nurses. The type of MHCUs admitted included assisted and involuntary MHCUs with different diagnoses such as substance use disorder, schizophrenia and bipolar mood disorder.

### 2.3 Sampling

A face‐to‐face recruitment method was used to purposively select participants. The research was introduced to potential participants in different wards during their lunchtime break. Potential participants were asked to call or message the researcher after which a brief interview was conducted to determine if the potential participant met the following inclusion criteria: Nurses with a psychiatric qualification, who responded to an invitation to participate in the study and provide care to MHCUs with dual diagnoses in a psychiatric hospital. Professional nurses with < 2 years of experience in the study context and enrolled and assistant nurses were excluded from the study. The researcher selected participants who met the inclusion criteria regardless of their gender.

The sample size was determined by data saturation (Polit and Beck 2021) when no new information emerged during interviews. The first author, a male psychiatric nurse, interviewed 10 participants after which no new information emerged and two more interviews were done to ensure data saturation was reached.

### 2.4 Data Collection

Individual face‐to‐face, semi‐structured interviews and observational, methodological and personal field notes (Polit and Beck 2021) were used to collect data from the psychiatric nurses caring for relapsed MHCUs with dual diagnoses at a public psychiatric hospital. The participants were interviewed by the first author (a male psychiatric nurse employed in the study context) in English between April and June 2020 in a private office in the hospital where they felt comfortable. A pilot interview was conducted with one participant who was not involved in the actual research and this assisted in determining the feasibility of the selected research methods, procedures and research questions.

The interviews were conducted during lunchtime or after work before participants left the premises of the institution. The participants signed informed consent before the interviews which lasted 45–60 min, depending on data saturation. After establishing rapport, participants were asked about the barriers they experienced to facilitating therapeutic relationships with MHCUs with dual diagnosis. This was followed by open‐ended probing questions. Interviews were audio‐recorded with the permission of the participants. The interviewer also took field notes to note communication dynamics and non‐verbal cues during and after the interviews to increase the credibility of the data.

### 2.5 Data Analysis

Interviews were transcribed verbatim which ensured familiarity with the data by listening to the recordings and re‐read the transcripts and field notes. Data were analysed thematically by the first author and an independent coder following the six phases of Braun and Clarke (2023: 65), namely, familiarisation with the data; generating initial codes; searching for themes; reviewing themes; defining and naming themes and producing the report. The coded data constituted the subthemes which were clustered into themes based on similar meanings. Transcripts were also analysed by an independent coder, with experience in qualitative studies and a consensus meeting was held with the independent coder to verify the themes and subthemes.

### 2.6 Trustworthiness

Lincoln and Guba's criteria of trustworthiness, which include credibility, transferability, dependability and confirmability (Polit and Beck 2021), were applied to ensure the rigour of the qualitative data. Credibility was achieved through prolonged engagement and spending sufficient time collecting data and taking field notes to enrich the data. Triangulation was ensured by interviewing nurses from different shifts and units. To ensure member checking, the researcher verified findings by going back to the participants to verify the themes and subthemes.

Transferability was ensured by providing a comprehensive description of the methods and the use of purposeful sampling to ensure that participants with information regarding barriers to therapeutic relationships with MHCUs with dual diagnoses participated in the study. Dependability was achieved through rich and detailed descriptions and the use of a co‐coder, a lecturer experienced in qualitative methods who added a different perspective to the interpretation of the findings. A consensus discussion between the researcher and independent coder served to confirm the accuracy of the findings.

### 2.7 Ethical Considerations

Permission to conduct the study was obtained from the University of Pretoria Ethics Committee (673/2020) and the management of the psychiatric hospital and provincial authorities. All participants voluntarily participated in the study after signing an informed consent form that explained the objectives and methodology of the study. Participants were also informed that they had the right to withdraw from the study at any time without any consequences. No participants withdrew from the study. Confidentiality was ensured by the use of code names in the reporting of findings.

## 3 Findings

### 3.1 Demographic Profile of Participants

The sample consisted of 12 psychiatric nurses who provided care for MHCUs with dual diagnosis. The three male and nine female participants were from the Vatsonga cultural group and their ages varied between 25 and 55 years old. Years of experience at the psychiatric institution ranged between 5 and 20 years. See Table 1.

**TABLE 1:** Demographic profile of participants.

Criterion	Characteristics	Frequency
Gender	Male Female	3 9
Ethnicity	Vatsonga cultural group	12
Experience	5 years 6–10 years 11–20 years	2 7 3

Table 2 summarises the themes and subthemes that were identified during data analysis. The two main themes are the barriers to facilitating therapeutic relationships and the recommendations of nurses to improve the relationship. All themes have subthemes and are discussed in detail evidenced by verbatim quotations in italics. The number of participants is indicated in brackets after the quotation.

**TABLE 2:** Overview of the themes and subthemes.

Themes	Subthemes
MHCU‐related barriers to facilitate a therapeutic relationship	MHCUs' unmanageable and disruptive behaviour. MHCUs' disrespectful behaviour. MHCUs' access to substances. Ambivalent nurse–patient relationship
Recommendations to facilitate a therapeutic relationship	Acceptance to cope with challenging behaviour and maintaining a therapeutic relationship. Support from the interprofessional team Empowerment of nurses

### 3.2 Theme 1: MHCU‐Related Barriers to Facilitate a Therapeutic Relationship

In this theme, four subthemes emerged, namely MHCUs' unmanageable and disruptive behaviour, MHCUs' disrespectful behaviour, MHCUs' access to substances and ambivalent nurse–patient relationship.

#### 3.2.1 MHCUs' Unmanageable and Disruptive Behaviour

The participants reported difficulties in creating and maintaining a therapeutic relationship with MHCUs who displayed manipulative and aggressive behaviour towards nurses and other MHCUs. MHCUs sometimes demand discharge from the hospital and refuse to take their medication. In an attempt to be discharged from the hospital, MHCUs may disrupt the routine, refuse meals, and project their anger towards other MHCUs as explained in the next quotation:

…their behaviour is unacceptable. They refuse medication, and then they tell us that if we don't discharge them they are not going to continue taking their medication. And when we think it's a joke, it becomes serious. We give them medication, they throw it away, and they disrupt every activity…they start to be uncooperative, they go to the extent of a hunger strike, and they refuse meals: ‘I'm not going to eat, as long as you are still keeping me here’. They are very intrusive…they are frequently demanding the same things all over again…they provoke us… if we don't give them attention, they divert their anger towards other patients… (P/5).

Some MHCUs are ‘always angry’, restless and resist hospital treatment. They threaten to harm themselves, other MHCUs or mental healthcare professionals when they meet them in the community after discharge:

…you will find MHCUs roaming around, pacing up and down…‘If you don't discharge me…. when I meet you outside in the mall, I will kill you or maybe I will injure someone here or maybe will even, kill myself here in the hospital to show you that I want to go home’. The MHCUs are always angry… (P/7).

When faced with constant anger and demanding behaviour, nurses find it difficult to provide care and feel powerless as their best efforts to engage MHCUs seem to be fruitless. Fear of repetition of physical or verbal aggression leads to avoiding contact with certain MHCUs for self‐preservation as evident in the next descriptions:

…they also fight us as nurses, if they want something and don't get it. If a patient wants to smoke, and then doesn't get their way, they become aggressive, and when you talk to them, they become angry with the nurses which is not a good thing to us as nurses. It's difficult for us to nurse them (P/11).

…when they beat the nurses or insult us, they think we don't treat them well, meanwhile we are trying our best to engage them in all activities. They say that they have a right to do this and this, a person who displays aggressive behaviour could possibly kill us…a MHCU insulted me in a way that I was even afraid to look at him the following day fearing that he would do the same again… (P/12).

#### 3.2.2 MHCUs' Disrespectful Behaviour

Participants felt that the MHCUs' disrespectful attitudes, especially towards female nurses affect the therapeutic relationship. Nurses feel disrespected when MHCUs refuse treatment and do not want to follow the ward routine such as participation in self‐care activities.

…so as female staff they don't respect us, they insult us…they refuse to take treatment sometimes because they are males…they argue when we nurse them…they don't listen to us when we tell them to go and bath and they say a lot of things…in fact, they don't respect us (P/12).

Female nurses are threatened with rape and sometimes inappropriately touched by MHCUs; such actions have emotional effects such as anger, fear and sadness. Participants are aware that angry responses towards MHCUs may lead to disciplinary action, therefore they feel powerless:

We see them attacking our fellow nurses or even attempting to rape them…threatening them… they are verbally aggressive, they are physically aggressive…they insult us, they threaten to rape us… (P/4).

…the patients disrespect us…the patients provoke us, some just touch us unnecessarily, inappropriately, especially as women, and when we become angry, we are suspended so that we are not comfortable. Even though we enjoy working in this institution in psychiatric wards, but the treatment that we get makes us sad… (P/6).

The disrespectful behaviour of MHCUs is met with some sort of retaliation, for example, in the next quote, some MHCUs are refused favours such as using nurses' cell phones to call their families:

…then there are those patients we won't assist with some personal requests like if they can talk to their parents because they are not allowed to have their phones…so for such MHCUs, we won't go the extra mile of taking our phones to say, you can you use our airtime because we know that MHCU is very disrespectful (P/7).

#### 3.2.3 MHCUs' Access to Substances

Participants were discouraged by the fact that many MHCUs continued to use substances in the ward. Nurses feel powerless to control access to substances as their enquiries regarding the origin of the ‘smuggling’ are met with denial and secrecy that result in distrust in the nurse–patient relationship:

…sometimes they hide the marijuana and smoke inside the ward when they want. When we try to find it, they fight us and say they don't have anything, they are not smoking, but they smell of dagga [cannabis]. They refuse to answer us and give a clear explanation as to where these drugs come from or where they found them (P/2).

The presence of substances in the ward causes suspicion and distrust among staff members. Participants suspected that some staff members used and provided substances to MHCUs which compromised professional nurse–patient relationships:

We have some people in the hospital who also use substances, and some of them smoke, so when the MHCUs see them smoking they crave…at the end of the day they want these substances. They end up making friends with these people (P/3).

Family members also reported that MHCUs are using substances in the hospital, causing more suspicion and self‐doubt among nurses:

Also, family when they visit them, they find them smelling of substances…it looks as if psychiatric nurses are part of the problem…like maybe we are the ones smuggling these substances like we are incompetent, but we don't know how they come to be in possession of these substances while hospitalized (P/4).

There are no supportive interventions from management when MHCUs are found to have substances. The psychiatric nurses felt powerless to address the access to substances in the ward as exemplified in the following quote:

No corrective measures are taken when the patient is found with substances, so any effort we make as psychiatric nurses, is in vain because they know that we take the substances away today…they will get more tomorrow, and nothing will happen to them… (P/7).

When nurses detect substances in the ward, the MHCUs in possession threaten to retaliate causing fear and hostility in the nurse–patient relationship:

…but of course, some [nurses] do not cope properly with…these challenges particularly those [nurses] who have found these MHCUs in possession of substances they live under fear that those…the same MHCUs they reported or took the substances from them may attack them…and then sometimes it creates a permanent enemy between psychiatric nurses and MHCUs (P/1).

#### 3.2.4 Ambivalent Nurse–Patient Relationship

This subtheme reveals how the abovementioned barriers affect the nurse–patient relationship, causing ambivalent feelings. While nurses know that have to maintain the ingredients of a therapeutic relationship, the relationship is sometimes characterised by distrust and hostility, especially shortly after admission when the MHCU is still aggressive and delusional. Nurses are seen as a threat who block MHCUs' access to the substances that they crave. When MHCUs recover they realise that nurses acted in their best interest, but with some MHCUs, the tension in the nurse–patient relationship never subsides and ‘becomes broken permanently’ as described below:

Most of them are addicted so when you try to stop an addict…from taking the very same substances you might experience serious challenges, so the relationship sometimes becomes sour and very tense. With some it's good, particularly with those who recover and realize that your effort in trying to stop them from taking the substances is in their best interest, so there might be a good relationship, but with some, it becomes broken permanently… (P/7).

MHCUs feel that they have a right to use substances and project their anger and hostility towards the nurses who prevent them from using substances.

Well, the relationship always has to be professional but sometimes we deal with MHCUs who personalize the relationship, particularly when they feel we are denying them their right. They think it's their right to smoke, and to use substances, so if we try to prevent them from taking the very same substances, we become the target…to them, we become the enemy when trying to assist them, so it becomes a very hostile relationship at times… (P/3).

Participants know that they are supposed to establish a positive and trusting relationship that fosters empathy and facilitates the healing effects of therapeutic methods:

…the relationship between the psychiatric nurses and the patients should be positive …so that we can win him or her over so that in the end trust is developed. It's then easy to conduct therapy sessions because we are positive…showing empathy or sympathy towards their condition… (P/4).

When the MHCU recovers the relationship is characterised by positive communication and a feeling of being grateful. But always present in the mind of nurses is the understanding that the relationship will be tainted again when the MHCU is readmitted:

Then, by the time they are discharged we are relating well with them, we have a good relationship because they are now able to thank us as staff, as health care providers…and we communicate well because now they are stable, but when they come back, they think we are giving them poison and…they are so difficult… (P/8).

### 3.3 Theme 2: Recommendations to Facilitate a Therapeutic Relationship

The following three subthemes emerged from this theme: acceptance to cope with challenging behaviour and maintaining a therapeutic relationship, support from the interprofessional team and empowerment of nurses.

#### 3.3.1 Acceptance to Cope With Challenging Behaviour and Maintaining a Therapeutic Relationship

One of the participants explained that aggressive behaviour can cause ‘bitterness’ and affect the therapeutic relationship, but nurses have to understand and accept that the MHCU's behaviour is related to the effects of substance abuse. Acceptance and tolerance are described as part of professional growth:

…obviously, the bitterness from me will be there, and then because I have got a passion to assist, I realize that this person is not doing it on his own, it's because of these substances, so sometimes, I can tolerate…so the relationship will start being bitter, but because I'm coming across him or her every day it means obviously I will have to understand and as a professional have to grow to help the patients…to accept even if we have bitterness… (P/2).

Nurses need to accept that they cannot help all MHCUs, but do their best to cope with the challenging behaviour of MHCUs without ‘hardening’ themselves:

…the first thing is to make peace that you cannot help everyone and then you can do what you can with what you have and not harden yourself, so do your best trying to cope with the situation… (P/7).

For other nurses, it may take a few weeks to come to terms with or cope with being insulted or threatened by a MHCU:

…coping with these challenges…I can say, I'm able to cope with them but it's not simple because after…being insulted by a person or after a person threatened to hit you, the following day when you look at that person, all those things that he did to you…he can still do it again…so it's very difficult to cope…too me it's not simple but after some, maybe a few weeks, I manage to cope but it's not simple… (P/9).

The same participant mentioned that nurses should understand that managing MHCUs with aggressive behaviour is part of their work and that it should not affect the nurse–patient relationship:

I can be able to understand that it is part of my work…I will be able to understand that after being insulted by a patient or after a patient has hit me that doesn't mean that our relationship should change because at that time the patient was not stable… (P/9).

The basic principles of maintaining the therapeutic relationship were mentioned by participants as follows:

…we don't judge them, we just support them unconditionally, I think it would be better… (P/11).

#### 3.3.2 Support From the Interprofessional Team

Participants indicated that they needed support from the interprofessional team to build relationships with MHCUs. Collaborative interviews with MHCUs can help to determine the reasons for substance use and explore alternative coping skills. The team should meet and set objectives for each MHCU and plan interventions to support the MHCUs. The next quotations provide evidence.

…and so together as MDT [multidisciplinary team] we are coping, the patient is interviewed and then we talk about the reason for using substances and how can they cope if they have personal problems so that they are able to manage their stress or life problems rather than solving their problems in the way of using substances… (P/7).

…the multidisciplinary team must sit down and must discuss…have the objectives of what it is exactly that they want to achieve in helping MHCUs who have substance abuse and how are they going…the strategy behind that, how are they going to come up with activities to meet the objective… (P/8).

Participants need resources such as referring MHCUs to a psychologist if they feel that the nurse–patient relationship is no longer conducive. At the time of the research, there was no psychologist appointed at the clinic.

…maybe if they can refer that person to a psychologist where they can get counselling…I think it can be helpful for that person because after coming across relapsed MHCUs, I think a nurse sometimes sort of…even the relationship between me and the MHCU is no longer the same… (P/9).

#### 3.3.3 Empowerment of Nurses

Participants indicated that they needed some form of empowerment to strengthen their relationship with MHCUs. The management of the hospital needs to visit the wards and provide education and support to nurses:

…they come in every morning to check on the patients and on the staff and sometimes give education to the staff as management. I know they have more knowledge than us, if they come and sit with us and give support and talk to us and want to know how are we feeling and how are we coping with this situation and give us advice on the management of the patients maybe in that way you can be able to be free in dealing with the patients… (P/6).

In‐service training, self‐empowerment and support from colleagues were mentioned as other ways to empower nurses so that they do not lose hope or keep a grudge against a MHCU:

I think if we educate each other regularly in in‐service training…remind each other…when you come across such a person this is what you do…some people they can forget…maybe you have been taught things ten years back…we have to support each other…we equip each other with recent knowledge…support each other how to manage MHCUs… (P/7).

…they [nurses] can empower themselves by reading or googling on how to take care of MHCUs…so that they [nurses] must not lose hope…they [nurse] must know that those people are mentally ill…they [nurses] must not keep a grudge…they [nurses] must be patient…and we will be proud of their [MHCUs'] success of one or two of them…they [nurses] will say one day, I treated this patient but now he is the manager at this company… (P/12).

Nurses should receive counselling to deal with the trauma of being exposed to or having witnessed violent assaults of colleagues or other MHCUs. At the time of the research, nurses received no counselling or debriefing as evident in the next two quotes:

I think counselling can help some of the professional nurses to understand that and another thing that can even boost the morale of a nurse in this situation… (P/9).

…to help you deal with the trauma because it's very traumatizing, we need some counselling, you know just someone to talk to overcome these because it's not nice to be attacked, it's not nice when you see your patient being attacked…I saw nurses being attacked in front of me and I was also attacked, and you know nothing happens at all… (P/4).

## 4 Discussion

The findings described the barriers to establishing therapeutic relationships with MHCUs with dual diagnosis such as disruptive and disrespectful behaviour and substance use during hospitalisation that result in a nurse–patient relationship characterised by ambivalent feelings. The participants made recommendations for nurses to improve the nurse–patient relationship such as acceptance, support from the interprofessional team and empowerment of nurses.

The disruptive behaviour of MHCUs is a significant barrier to maintaining therapeutic relationships. Patients with psychotic disorders and substance abuse disorders present a high risk for violent behaviour and providers should be aware of their feelings, prejudices and fears to maintain empathy with the MHCUs (Uchtenhagen 2020). The literature highlighted various challenges associated with both mental illness and substance use that may disrupt the therapeutic relationship. During psychiatric hospitalisation symptom severity, involuntary admissions and coercion may affect the nurse–patient relationship (Bolsinger et al. 2020). These challenges are compounded by aggressive behaviour (Uwalaka 2023) and impulsivity due to substance use disorders, the latter also affecting treatment completion (Daigre et al. 2019). Restorative justice practices are recommended in some studies (Martin et al. 2023) to address violent behaviour and help patients and nurses cope with the aftermath. Sharing of emotions and vulnerability may create an opportunity for MHCUs and nurses to restore the therapeutic relationship.

Psychiatric nurses felt discouraged when MHCUs used substances in the ward and some even indicated that they were no longer interested in stopping the MHCUs from using substances. Strike et al. (2020) found that patients used psycho‐active substances during hospital admissions for reasons such as substance withdrawal symptoms, boredom, sadness, loneliness and untreated pain. As in the current study, patients concealed substance abuse from healthcare professionals. When healthcare professionals detect substance use, they respond in different ways such as ignoring it, increasing patient observation, reprimanding patients and threatening patients with discharge (Strike et al. 2020). Specific to psychiatric settings, professionals felt unsure when MHCUs concealed their actual level of substance use (Petersen, Thurang, and Berman 2021). Relapses in the use of psychoactive substances harmed the therapeutic relationship and professionals described users with substance abuse as having a ‘rebellious attitude’ (Martins Sousa et al. 2023). When nurses are required to take a custodial role, it may cause tensions in the therapeutic relationship (Hurley et al. 2022), for example, aggression precipitated or exacerbated by nurses' efforts to control access to substances, a function that can rather be fulfilled by security staff.

In this study, participants wished to overcome the barriers to building and maintaining recovery‐focused therapeutic relationships, which is a constantly evolving process as indicated by Moyles, Hunter, and Grealish (2023). Acceptance is a component of the therapeutic relationship where nurses provide support to MHCUs so that they can feel understood as a person. Nurses should be competent in relating with and accepting MHCUs to facilitate the recovery process and protect themselves from professional burnout (Aznar‐Huerta et al. 2021). From patients' perspectives, helpful nursing interventions include education about the side effects and impact of substances on mental health, listening and demonstrating respect, honesty, kindness and compassion. The therapeutic relationship should acknowledge the unique needs, priorities and preferences of each MHCU (Brahim, Hanganu, and Gros 2020). Establishing therapeutic communication requires nurses to be honest, empathetic, and self‐aware. Self‐awareness includes awareness of limitations (Heydari and Mirhaghi 2023) and to ‘make peace that you cannot help everyone and then you can do what you can’ as said by a participant in the current study.

Nurses who participated in this research expressed a need for interprofessional support and training to help them cope with the barriers they experience. Studies recommended an interprofessional and integrated therapeutic approach to enhance recovery for MHCUs with dual diagnosis (Daigre et al. 2019). Interprofessional team‐based mental healthcare enhances provider and nurse satisfaction and reduces provider burnout and the average length of hospitalisation (Oldham et al. 2020). Buljac‐Samardzic, Doekhie, and van Wijngaarden (2020) identified interventions to improve teamwork such as training and organisational structures to stimulate team processes and team functioning. Training in dual diagnosis led to significant improvements in the knowledge, attitude and self‐efficacy of healthcare professionals in a hospital in Canada (Chicoine et al. 2023) and improved treatment at Danish mental health centres (Pinderup 2018).

### 4.1 Strengths and Limitations

All psychiatric nurses in the study were from the Vatsonga cultural group meaning that other culture groups were not represented. Most of the participants were females which is reflective of a largely female nursing profession. Participants who contacted the researcher were selected regardless of their gender. Other mental healthcare practitioners were not included in the study. The study was done in a poor resource area which might have contributed to participants' challenges. The homogenous study sample could have yielded skewed results. The results cannot be generalised to other categories of nurses and settings due to the small sample size.

A strength of the study is that it not only provided insights into nurses' problems but also highlighted their recommendations to resolve the problems from their perspectives. The findings were communicated to the management of the hospital to strengthen support and training for the nurses.

### 4.2 Implications for Practice and Research

Nurses recommended the strengthening of interprofessional mental healthcare practice, improved teamwork, training opportunities and support from management. There is a need for a clear policy regarding actions to be taken when MHCUs use substances in the hospital. Security policies and measures should be in place to prevent substances from entering the hospital and protect nurses when MHCUs present with aggressive and unacceptable behaviour. Nurses who were exposed to aggression need counselling and debriefing to help them manage negative feelings. Upgrading facilities to ensure safe infrastructure, sufficient human resources (including male psychiatric nurses) and acknowledgement of psychiatric nurses' contributions may help to boost their morale to provide effective mental healthcare. Ongoing training of nurses to maintain therapeutic relationships, manage disruptive behaviour effectively and understand and manage MHCUs with dual diagnosis may help to empower nurses who are struggling to maintain caring relationships in ‘non‐caring’ circumstances.

Action research to implement strategies to empower and support nurses is recommended. Reflective cycles may help to refine interventions until nurses feel competent to manage MHCUs with dual diagnosis. Quantitative research to explore barriers in different psychiatric settings may help to understand the extent of nurses' challenges in managing patients with substance use problems.

## 5 Conclusion

The psychiatric nurses highlighted their experiences in establishing therapeutic relationships with MHCUs with dual diagnoses. Barriers included MHCUs being aggressive, unmanageable, disruptive and disrespectful towards psychiatric nurses and access to substances. All these challenges affect the therapeutic relationship between psychiatric nurses and MHCUs and result in ambivalence towards MHCUs. Nurses realise that they are required to show acceptance towards MHCUs and tolerate angry behaviour to not affect the therapeutic relationships, even if it is difficult at times. The psychiatric nurses expressed a need for support from the interprofessional team and management to overcome these barriers.

## Author Contributions

T.M., A.E.W. and T.I.R. were involved in the conception and design of the research project. Data collection and analysis were done by T.M., A.E.W. and T.I.R. revised the article content and approved the version to be published.

## Ethics Statement

Ethical approval was obtained from the Research Ethics Committee, Faculty of Health Sciences, University of Pretoria, Ethics reference no: 673/2020.

## Conflicts of Interest

The authors declare no conflicts of interest.

## Data Availability

The data supporting the findings of this study are available from the authors.
